# Mature Spinal Teratoma: A Case Report

**DOI:** 10.7759/cureus.53785

**Published:** 2024-02-07

**Authors:** Jorge Del Pino-Camposeco, Eliezer Villanueva-Castro, Obet Canela-Calderon, Juan Antonio Ponce-Gómez, Noe Alejandro Salazar Felix, Juan Nicasio Arriada-Mendicoa

**Affiliations:** 1 Department of Neurosurgery, Instituto Nacional de Neurología y Neurocirugía Manuel Velasco Suárez, Mexico City, MEX; 2 Department of Neurosurgery, Instituto Nacional de Neurología y Neurocirugia Manuel Velasco Súarez, Mexico City, MEX

**Keywords:** cauda equina tumor, melanocytic content, surgery spine, benign mature cystic teratoma, primary spinal tumor

## Abstract

We presented an unusual case of a teratoma in a 76-year-old female who began four years ago with paresthesias and hypoesthesias in the sacral and gluteal regions. She denied weakness or gait instability. The magnetic resonance imaging showed an intradural lesion within the cauda equina at levels L2-L3. We decided to perform a posterior midline approach to the lumbar region to expose L2-L3 levels. After doing the L2-L3 laminectomy and the durotomy, we found a solid lesion surrounded by nerve roots with heterogeneous content. Through the meticulous separation of the nerve roots surrounding the lesion, we punctioned it, observing the exit of melanocytic material. Histopathological findings showed germinal neoplasia without immature neuroepithelium or malignant component; therefore, the diagnosis of mature teratoma was made. The patient was discharged without any aggregate neurological deficit. At the six-month follow-up visit, the patient continued with paresthesia in the gluteal region without motor weakness and reported minimal gait improvement.

## Introduction

Central nervous system (CNS) teratomas are infrequent; in the majority of cases, they are intracranial, located in the pineal gland, posterior fossa, cerebral hemispheres, and suprasellar region [1]. True teratomas are classified as tumors generated from all three germ layers; teratomas are tumors of germ cell or dysembryogenic origin that arise from the ectopic development of two or more totipotent cell lines (ectoderm, mesoderm, and endoderm) [2,3]. Based on the degree of differentiation, teratomas are classified into three: malignant, immature, and mature. The origin of spinal cord teratoma is controversial; misdisplacement of multipotential germinal cells is the most accurate theory [4]. Teratomas can be found throughout the spine, with more prevalence in the thoracic and lumbar regions and most frequently in the conus medullaris [5]. Despite the fact that these tumors can appear in various locations, teratomas make up less than 1% of CNS tumors and are extremely rare in the spinal cord [6]. Since teratomas have an unpredictable natural progression, total surgical removal is often the main primary treatment [7]. Symptomatic recurrence in mature teratomas is uncommon, even in cases with subtotal resection, The recurrence rate has been reported at about 10%, predominantly seen in immature and malignant forms [8].

## Case presentation

**Figure 1:**
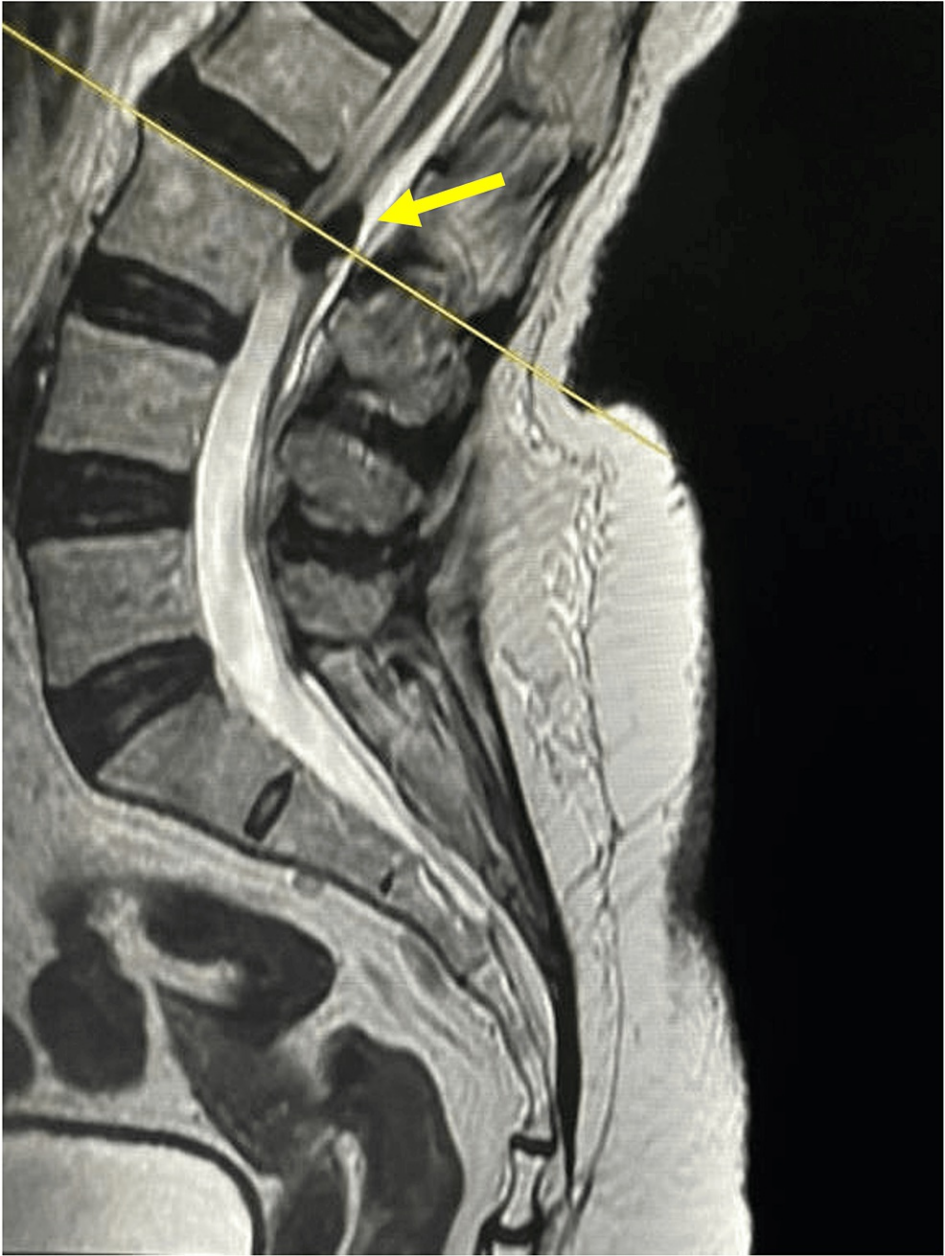
Preoperative imaging studies A sagittal T2 sequence MRI shows a tumor in L2-L3 intervertebral space (yellow arrow).

The case of a 76-year-old female patient with no significant personal pathological history is presented. Her condition began four years ago with paresthesias and hypoesthesias in the sacral and gluteal regions, irradiation to the right pelvic limb, and stress urinary incontinence. She denied weakness or gait instability. On the neurological examination, the patient was conscious, alert, and oriented in person, time, and place. Mental functions and the cranial nerves were preserved. Cerebellum without pathological findings. Strength was 5/5 in the upper limbs and 4+/5 in the lower limbs based on Daniel´s strength scale, proximal and distal. The sensation was intact to pinprick, vibration, and proprioception; she had hypoesthesia in L4 distribution in both lower limbs with a light touch. Muscle stretch reflexes were ++ in the upper and lower extremities. Bilateral flexor plantar response. Magnetic resonance was performed in T2, T1, and fat suppression sequences, which showed a Hypointense lesion inside the spinal canal without foraminal or paravertebral extension within the cauda equina at levels L2-L3 (Figure 1).

**Figure 2:**
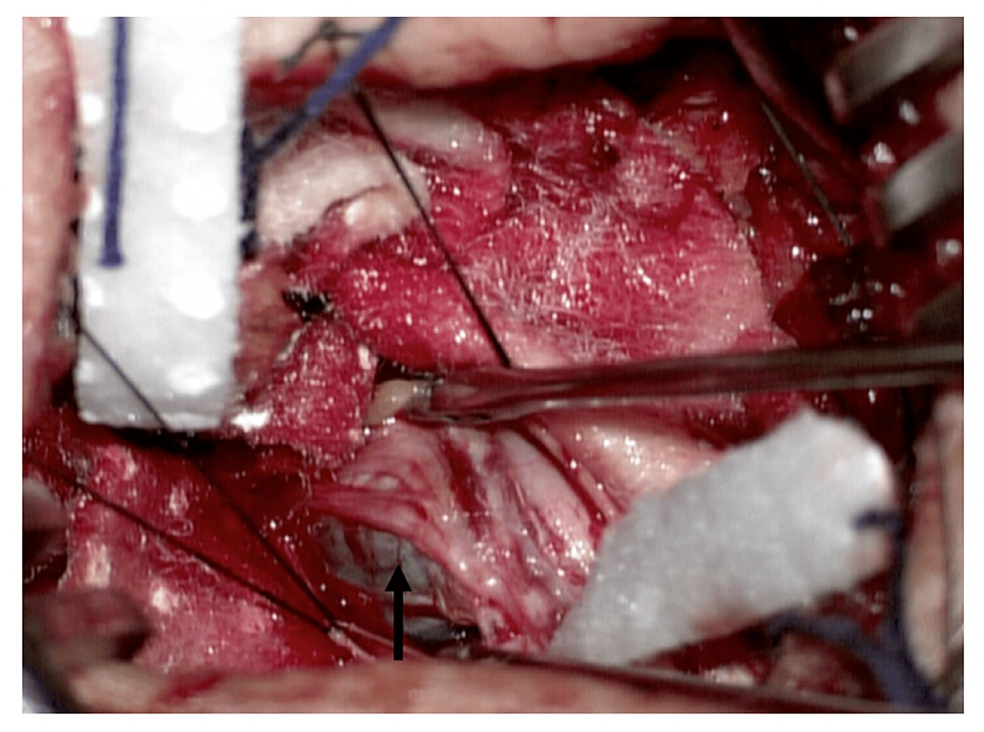
Transoperative image one The tumor was involved in the nerve roots of the cauda equina(black arrow).

**Figure 3:**
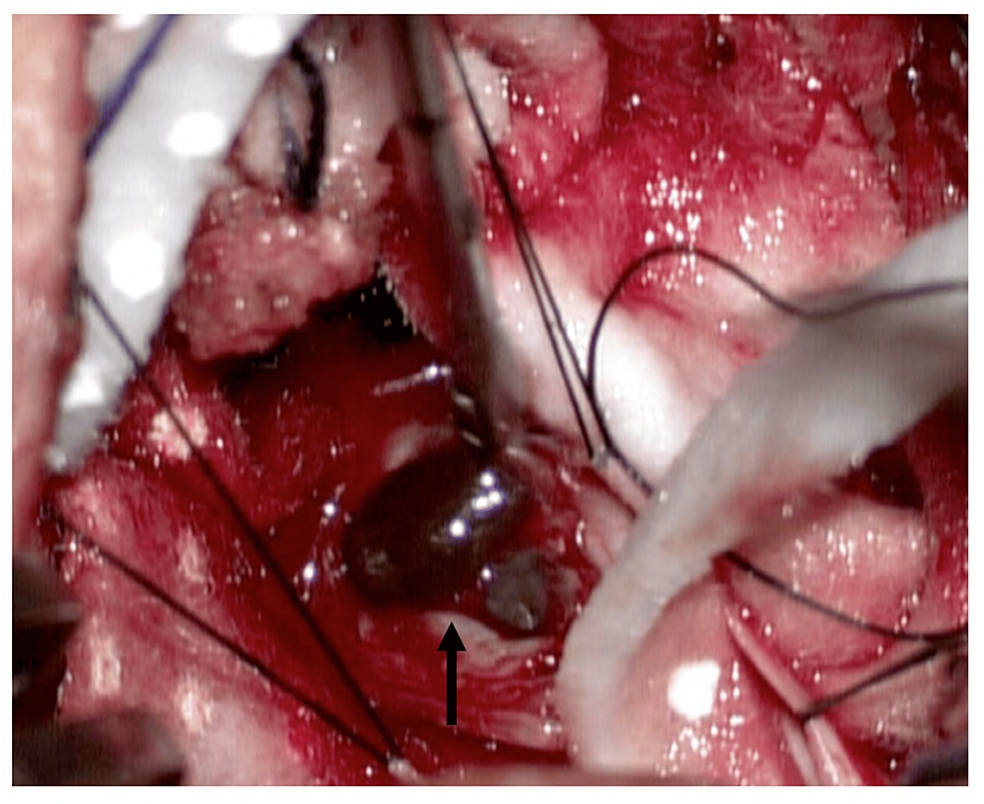
Transoperative image two The tumor after separation of nerve roots and puncture with the exit of the melanocytic component (black arrow).

Based on clinical and image findings, we concluded this lesion was a spinal intradural extramedullary tumor to rule out a neurofibroma or schwannoma. We decided to perform a posterior midline approach to the lumbar region to expose L2-L3 levels. After doing the L2-L3 laminectomy and the durotomy, we found a solid lesion surrounded by nerve roots with heterogeneous content (Figure 2). Through the meticulous separation of the nerve roots surrounding the lesion, we punctioned it, observing the exit of melanocytic material (Figure 3). After aspiration of all the contents, the residual lesion was separated from the nerve roots and completely resected. After three days of intrahospital recovery, the patient was discharged without any aggregate neurological deficit. At the six-month follow-up visit, the patient continued with paresthesia in the gluteal region without motor weakness and reported minimal gait improvement.

Histopathological findings

**Figure 4:**
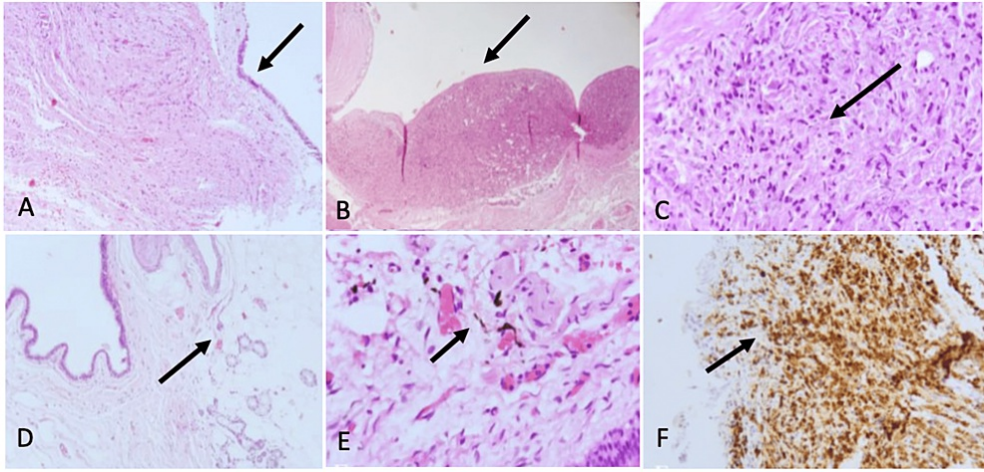
Histopathological findings (A) Panoramic view of a cyst delineated by epithelium. The cystic wall is made of fibrous and nervous tissue (H&E; 4X) (black arrow); (B) Nodular and disorganized proliferation of cells from ectodermal and mesodermal origin (H&E; 4X) (black arrow). (C) A nest composed of fusiform cells with hyperchromatic nuclei and some pigment inside the cytoplasm (H&E; 40x) (black arrow). (D) Cyst wall with ectodermal components, adipose tissue and glands (H&E; 4x) (black arrow). (E) The epithelium is columnar, pseudostratified with cilia, with respiratory phenotype. Some pigmented fusiform cells can be seen (H&E; 40X) (black arrow). (F) Cells of the nodular proliferation showed Melan A immunoreactivity (10X) (black arrow).

Germinal neoplasia is obtained through histological sections with the presence of cellular components from the three germ layers. A cystic area is observed covered by ciliated columnar epithelium; in some areas, it is pseudostratified with goblet cells. Basement membrane and fibrous or subepithelial tissue with variable degrees of cellularity, the presence of cerine glands, mature adipose tissue, and smooth muscle fibers are observed. Irregular and tortuous nerve tracts are observed, and some spindle cells show melanin pigment. Areas with mature brain tissue are identified. No immature neuroepithelium or malignant neoplasm is identified (Figure 4).

## Discussion

Spinal teratomas are uncommon lesions. They account for only 0.2-0.5% of all spinal cord tumors [5,9]. The clinical presentation of adults is highly variable depending on the location of the tumor in the spine (cervical, thoracic, or lumbosacral) and whether the tumor is intramedullary, intradural, extramedullary, or extradural [5]. Mature intraspinal teratomas in adults are generally slow-growing, located generally between the lower thoracic region and the lower thoracic medullary conus [10]. The patient in our case only had a mild motor deficit that limited ambulation without pain, and the lesion was located at the beginning of the cauda equina in L2-L3.

Both mature and immature teratomas are generally benign, particularly when promptly treated. Mature teratomas contain components that are completely differentiated. Immature lesions usually contain fetal tissue. Malignant teratomas have the malignant component of the germ cell layer [5]. In the case presented, the histopathological findings showed areas with mature brain tissue. No immature neuroepithelium or malignant neoplasm was identified; therefore, the diagnosis of mature teratoma was made.

Teratomas are generally well encapsulated, have solid and cystic components, and show mixed signal intensities. Teratoma lesions have the following MRI image characteristics: well defined and they could have a thick wall circular or lobulated encapsulated cystic mass, hyperintense on T1 and T2 and hypointense images by the presence of adipose tissue on fat suppression sequences, calcifications, which mostly appear hypointense on all sequences, slight enhancement after contrast administration, sometimes associated with dysraphic congenital anomalies [11]. Recurrence depends on the histological features of the tumor; it is more frequently found in immature and malignant teratomas. However, symptomatic recurrence of mature teratomas is low, even in subtotal resections [2,5].

## Conclusions

Due to the benign nature of these tumors and their generally satisfactory long-term results with minimal recurrence rate, total surgical excision is the main action; nevertheless, it is advisable to do long-term surveillance. Although there are certain image characteristics that can suggest the presence of a spinal teratoma, the intraoperative and histopathological examinations provide the definitive diagnosis. It is unclear how adjuvant treatments, such as chemotherapy and radiation therapy, should be used for residual tumors. As demonstrated in this case, the majority of these patients may experience a very positive neurologic recovery.
